# “You feel like you going to die:” The intersection of mass incarceration and climate disasters

**DOI:** 10.1016/j.ssmqr.2025.100613

**Published:** 2025-08-09

**Authors:** Katherine LeMasters, Zaire Cullins, Erin McCauley, Lauren Brinkley-Rubinstein

**Affiliations:** a Division of General Internal Medicine, School of Medicine, University of Colorado Anschutz Medical Campus, Aurora, CO, USA; b Department of Epidemiology, School of Public Health, University of Colorado Anschutz Medical Campus, Aurora, CO, USA; c Department of Population Health Sciences, School of Medicine, Duke University, Durham, NC, USA; d Department of Social and Behavioral Sciences, University of California San Francisco, San Francisco, CA, USA; e Philip R. Lee Institute for Health Policy Studies, University of California San Francisco, San Francisco, CA, USA

**Keywords:** Incarceration, Disasters, Hurricanes, Environment

## Abstract

**Purpose:**

Incarceration is increasingly harmful for health due to climate hazards and the lack of policies to protect people who are incarcerated from flooding, hurricanes, and extreme temperatures. Yet, these overlapping crises have received limited attention. We assessed the experience of being incarcerated during hurricanes in Houston, Texas, an area repeatedly impacted by climate events. We conducted semi-structured interviews with 18 individuals accessing syringe exchange services in Houston, Texas. We then created templated summaries and matrices to conduct rapid qualitative analysis, an action-oriented approach to qualitative data analysis.

**Results:**

All participants had direct experience with climate disasters, most while incarcerated. Disasters often forced multiple people into small cells without power or potable water. Facility infrastructure worsened these experiences due to being partially underground and lacking adequate temperature control. Disasters highlighted individuals’ reliance on guards which contributed to high levels of uncertainty. Individuals were disproportionately impacted by hurricanes following community re-entry due to being unhoused, not being able to afford evacuation, and having work disrupted.

**Conclusions:**

Overall, hurricanes and other disasters imposed additional trauma on individuals living in stressful, unpredictable environments. Work at the intersection of the criminal legal system and health must consider how the climate crisis affects this relationship.

## 1. Background

Incarceration creates the perfect storm for mental and physical health crises due to overcrowded and unpredictable living environments, limited mobility, substandard healthcare, and the disproportionate levels of medical and behavioral comorbidities experienced by those behind bars. Health risks in carceral environments are increasingly compounded by exposure to climate hazards, as the number of climate disasters has increased fivefold in the past 50 years (World Meteorological Organization, 2022). Recent work has also conceptualized prison spaces as struggles for environmental justice as incarcerated individuals experience environmental racism and ecological injustice due to threats to human health that are already there (Pellow, 2021). The Prison Environmental Justice Project has raised numerous concerns regarding high degrees of toxic exposures in carceral facilities including water contamination and unsafe working conditions (The Prison Environmental Justice Project, 2020). Climate change will only exacerbate these existing environmental injustices in prisons and jails by causing more frequent and intense destructive hazards (Cowan et al., 2022; Glade et al., 2022).

These disasters are particularly common in Houston, Texas and the surrounding area, as this region increasingly experiences hurricanes, floods, poor air quality, and extreme heat (Petkova et al., 2015). Climate disasters have adverse impacts on health including increased infectious disease spread, chronic illness, physical injury, worsened mental health, and increases in substance use (Haines & Patz, 2004; Hayes et al., 2018; Reid et al., 2016). Given the increasing threats of climate change and the criminal legal system’s persistence, we expect this vulnerable population’s exposure to climate hazards to continually increase (Glade et al., 2022). Yet, little work has analyzed incarcerated individuals’ lived experiences of climate disasters and how such disasters impact health.

Further, research in Colorado found that approximately 75 % of incarceration infrastructure was already at a high risk for extreme heat, floods, wildfire, or landslides (Glade Sara et al., 2024). These many stressors are compounded by a lack of policies and practices (e.g., lack of air conditioning, dearth of evacuation policies) within facilities to protect incarcerated individuals from these hazards and pre-existing poor carceral infrastructure (e.g., outdated buildings) (Maner et al., 2022). There is also a lack of emergency management policies at the county and state level that include incarcerated populations. Prior work has documented emergency management policies for prisons under state and federal jurisdiction (Gaillard & Navizet, 2012), finding that evacuations are often deprioritized due to the focus on limiting escapes over preserving well-being (National Prison Project of the American Civil Liberties Union, 2006) and that only 17 of the United States 50 state prison systems have a published emergency management plan (Maner et al., 2022).

Prisons and jails are thus often conceptualized as spaces contributing to structural health inequities (Wildeman & Wang, 2017), an issue only exacerbated in a changing climate. Drawing on disaster studies, prisons and jails can also be conceptualized as spaces systemically subjected to the social production of risk. Incarcerated populations are particularly vulnerable to suffering from their heightened exposure to climate hazards given the growing and aging incarcerated population produced through the system of mass incarceration, a lack of policies and practices that protect individuals from climate hazards, and conditions of confinement that produce poor physical and mental health (Wisner, B et al., 2003). This high exposure to hazards and high vulnerability from suffering from exposure to such hazards are the two key components of the Pressure and Release Model that produce high risk situations (Wisner, B et al., 2003).

While prior work has thus established the lack of preparedness of these institutions for climate disasters and pre-existing environmental injustices, little work has analyzed incarcerated individuals’ lived experiences of climate disasters and how such disasters impact health. Reports published by the Marshall Project in the aftermath of Hurricane Harvey revealed a lack of communication to incarcerated individuals during a disaster and how the prison’s placement next to a reservoir posed an imminent threat when the reservoir filled with water (McDonald, D, 2018). While journalism has brought critical attention to these disasters, there is a need for a systematic qualitative exploration to more fully assess individuals’ experiences, to provide unique insight into how disasters are experienced by individuals with diverse backgrounds, and understand how policies are or are not implemented being carceral walls. Our objective was thus to assess how climate hazards affect the health of those with criminal legal involvement in Houston, Texas, an area repeatedly impacted by climate events.

## 2. Methods

### 2.1. Data collection

We conducted 60–90-min semi-structured interviews in Houston, TX with individuals utilizing syringe exchange services, a population disproportionately experiencing incarceration partially due to the criminalization of drug use (Wildra, E, 2024). Interviews focused on climate disasters, harm reduction services, and substance use. The research team collaborated with the Houston Harm Reduction Alliance, a community-based organization, to identify and recruit possible participants using convenience sampling. We received a waiver of written consent and obtained verbal consent. Institutional Review Board approval was granted by the University of North Carolina, Duke University, and the University of Colorado. Eighteen total interviews were conducted. Interview recordings were transcribed and anonymized, except for city names that were required to analyze impacts of natural hazards in a specific location. Participants received $50 in cash for participating in the interview.

### 2.2. Data analysis

Meaningful analytical units were analyzed using rapid qualitative analysis, often referred to as RQA, an action-oriented approach to qualitative data analysis when findings are quickly needed to inform policy (Vindrola-Padros & Johnson, 2020). Quality checks were undertaken to ensure high inter-and intra-coder reliability. First, templated summaries were created for each interview using Microsoft Excel. Each summary was organized by domains, or key topics, based on research objectives. One team member (KL) created summaries for each interview with a separate team member (ZC) reviewing each summary and associated transcript to confirm concordance. A matrix analysis was then conducted to present an organized display of summary data (Averill, 2002). To do this, ZC created summaries with example quotes, and team members then collaboratively identified and grouped key themes from the summaries. Numbers are used to differentiate participants throughout the results.

## 3. Results

Of the 18 participants, 10 self-identified as White or Caucasian, four as Black or African American, one as Hispanic and three as multiple racial or ethnic identities. Most (78 %) had received a high school diploma. Most participants were between 31 and 40. Thirteen respondents (72 %) discussed experiences of incarceration during a climate disaster.

In general, those interviewed described being incarcerated during climate disasters as scary and traumatizing because you “know that something might happen that’s very, very bad. I don’t want to die” (#1). These fears were both based on individuals’ own experiences and in anticipation of future events, with the same person noting that they had “heard all about it, it’s really, really devastating … when I was in [carceral facility] because the marks on the walls where the flood, where the water came up inside there” (#1). These events brought incredible amounts of uncertainty, with two individuals noting that it extended their periods of incarceration, with one individual recounting how “it was supposed to be overnight, and I ended up [incarcerated] for fucking 21 days” (#2).

### 3.1. Disasters forced multiple people into small cells without power or potable water: “You feel like you going to die”

Four participants described conditions of confinement during disasters as being crowded, hectic, and lacking basic sanitation. First, individuals would be tightly packed together, and “instead of having one or two people [per] cell, they packed each cell with seven, eight people” (#3). These conditions often lasted far past the disaster, with one respondent saying that “it took them 21 days … and you got two people in there … your arms’ width right now” (#2). These tightly packed conditions could lead to conflicts, because “when they were putting people in the cells like that, they didn’t know if they were putting people in with enemies” (#3). The crowdedness contributed to unsanitary conditions with people not having enough space to use the bathroom.

In addition to cells being crowded, two individuals noted having no water or only water that was non-potable. This - in addition to hurricanes being in summer months when individuals were already hot - prevented individuals from being able to shower for multiple days and not able to flush toilets when packed tightly together. Even if water was non-potable, this was often all individuals had to drink, and the guards “didn’t tell you, ‘can’t drink the water’” (#3), so individuals kept drinking it. Additionally, food supplies were sometimes cut off with incarcerated individuals only being brought food on the third day of a disaster.

During disasters, jails often lost power and that “no alarms would go off because they don’t want people to potentially be able to get out” (#4) leaving individuals confused. Without power, multiple participants stated that they could not call families or loved ones, as they could not and “didn’t use the phone at that time” (#2), contributing to more uncertainty and fear for how loved ones were coping.

### 3.2. Experiencing extreme temperatures during incarceration: “The walls sweat”

Six participants described being incarcerated during heat waves. These heat waves often overlapped with hurricanes, which one individual described as “a nightmare” (#5). This was exacerbated by facility infrastructure, with individuals describing how carceral facilities are built “just like big chicken coops, big metal buildings that just attract heat” (#6). Two participants discussed watching people die during heat waves - an additional participant discussed watching people die from flooding during a hurricane – and guards taking a long time respond, with one person stating “it’s actually the first time I’ve ever seen someone die right in front of me, this guy just, he died of a heat stroke … The nurses took an hour to get there or something like that and he just been laying there dead the whole time” (#6). On the opposite end of the spectrum, four participants discussed temperature swings, stating that “it was hot and it was extreme cold” (#7). While being incarcerated when it was extremely cold, individuals stated that they simply could not get warm. Conditions were described as being worse in solitary confinement, with one individual stating that it was “a concrete dungeon tomb … 25 feet underground” (#8).

### 3.3. Reliance on guards during disasters: “They’ll leave you in there”

Underlying the stress of being incarcerated during disasters was the behavior of guards, which four respondents discussed. During this time, one person stated that guards used a lot of force. Individuals acknowledged that while they were relying on guards for information, food, and medical attention, “they don’t know what the guards got going on too because the guards is in danger just as well” (#3). As normal routes for receiving food and medical attention were closed, guards were also waiting on emergency shipments. These individuals described feeling more vulnerable during this time, recognizing that “you could die there because if it floods, you’re behind bars. It is left up to the guards to get you, ok, if they can’t get you, you’re dying” (#1). This respondent expanded on this saying that as “water [is] coming up to your knees and you’re incarcerated, you’re in a cell, you can’t go anywhere and you’re waiting for the guards to come get you. They’re transferring you, but it is [going] slow, the lights are out” (#1). This particular reliance on guards during this time also interrupted the flow of transfers and releases from incarceration, leading to individuals being incarcerated for longer than anticipated. Some felt more negatively towards this situation, stating that if “the weather gets too bad, gets bad enough … they’ll leave you in there … [the guard] don’t want to save you” (#9). Relatedly, after disasters ended, they were treated by guards exclusively as sources of labor, with guards saying, “okay, this side is the wrecking crew, this side is the cleanup crew” (#8).

### 3.4. Consequences of climate disasters beyond carceral walls: “It’s hard to fucking make money”

Those who have spent time incarcerated were also disproportionately impacted by climate disasters during their time in the community. Systems-impacted people are disproportionately likely to be unhoused, work low-wage jobs, and work hourly jobs. Five participants described these factors as exacerbating the impacts of climate disasters during their time on the outside: they detailed not being able to afford to evacuate, and these climate events disrupting work. Individuals who were unhoused during disasters lost their car if they were trying to use it for climate control and often had to walk through storms when it was unsafe. If unhoused during extreme heat, people discussed how “people get agitated” (#2) when it is hot and humid and it makes people want to drink and be around criminalized substances that could put them back in jail. Overall, individuals recounted this experience as “scary … to be out there, they had no place to go, being homeless and taking on chance of dying, trying to survive” (#3). As individuals often could not evacuate, both respondents and their families “lost everything, their clothes got wet, shoes got swept away, shit got scattered and moved around … not having no property, not having no place to go and stuff like that. They already on the street so it affected them much worse than anybody else” (#3). Additionally, it was much harder to work in these situations, as people described panhandling for money or having informal employment.

### 3.5. Consequences of climate disasters beyond incarcerated individuals: “we were always the family that stayed back”

Climate disasters also affected the families of incarcerated individuals, compounding stress for those on the inside and eroding their support networks. While incarcerated, three individuals discussed being worried about family members because they “didn’t have money to evacuate, we had to wait this out” (#2) which was often amplified by power being out and not being able to check in via phone calls. Given that both incarcerated individuals and their family members were frequently in high-risk situations, people discussed “[having] a family member [who] was killed or something during the disaster” (#4), which could also lead people towards substances and increase their risk of overdosing. Individuals re-engaging in substance use to cope with these stressors then increased their risk of re-incarceration.

## 4. Discussion

Of 18 individual interviews conducted in Houston, Texas among individuals access syringe services, the vast majority, 13, had experienced incarceration during climate disasters, namely hurricanes and heat waves in Texas. In hurricanes, participants discussed feelings of stress, anxiety, and fear related to a lack of power, being crowded into cells, and a lack of food and water. In addition to poor conditions, individuals feared the unknown, not knowing if they would be evacuated, when they would receive food, and when they could check on family members. In heat waves, multiple participants noted watching individuals die of heat-related reasons, and others noted how the prison and jail infrastructure trapped in heat, making it unbearable. Underlying these experiences were tenuous relationships with jail and prison staff, noting how they were fully reliant on guards to improve conditions and provide information, a full loss of autonomy. These individuals also disproportionately experienced severe effects from climate disasters when not incarcerated due to being unhoused and reliant on informal work. Climate disasters also impacted the families of incarcerated individuals, disrupting communication and increasing stress. This was especially true given that participants were coming from families who were unable to afford to evacuate.

This work adds to the growing body of literature on (1) the experience of climate disasters in prison and jail settings and the impacts of hurricanes in carceral spaces specifically, (2) how climate disasters impact individuals with criminal legal histories even while not incarcerated, (3) the need for emergency management in prisons and jails, and (4) this work positions the criminal legal system as one that is integral to our understanding of the social production of climate-related risk with dire impacts for health. First, descriptive studies documenting climate-related exposures have found that the number of hot days per year increased during 1982–2020 for 1739 carceral facilities, primarily in the South (Tuholske et al., 2024). Recent qualitative work has documented individuals’ experiences with extreme heat and wildfires behind prison and jail walls, highlighting poor conditions, lack of access to resources, and tenuous relationships with guards (Barron et al., 2024; C. Purdum et al., 2024). However, little research has focused on patterns of hurricanes impacting carceral facilities. Journalism has been critical in documenting these experiencs, with the Marshall project documenting the experience of Hurricane Harvey in August 2017 behind prison walls (McDonald, D, 2018), and multiple outlets documenting the thousands of incarcerated individuals trapped in flooded cells without lights, running water, or any information for five days during Hurricane Helene in September 2024 (Mitchell, S, 2024). This work is a critical first step in documenting the lived experience of hurricanes - and how they often overlap with experiences of heat - in carceral settings.

Second, this work highlights that the continued punishment and harm that individuals face after incarceration (LeMasters et al., 2023; Phelps, 2013; Semenza & Link, 2019) is amplified by these individuals and their families facing disproportionate impacts of climate disasters. Similarly, a study of state parole policies and qualitative research in Texas, Louisiana, and Florida have found that community supervision policies do not address how individuals should respond during disasters and the implications for compliance failure, which could lead to reincarceration (Henry, F. & Wachtendorf, T., 2023; Henry & Wachtendorf, 2023a, 2023b). These disproportionate impacts on individuals and families under the purview of the legal system should serve as a call for both government entities and community organizations to focus on this population during disasters.

Third, our work highlights the lack of emergency management during hurricanes in prisons and jails. While most state prison systems lack emergency management plans for climate disasters, at least 30 states identify incarcerated individuals as surplus labor for disaster mitigation, preparedness, response, and recovery, exposing incarcerated individuals to hazardous conditions, often without sufficient personal protective equipment, little or no compensation, and no resources to cope with the stressors imposed (J. C. Purdum & Meyer, 2020). For example, incarcerated people were forced to install sandbags in Houston before Hurricane Harvey while the rest of the city evacuated and incarcerated people were not evacuated for several more days (Mosendz, P, 2017). This mismatch in emergency preparedness and response in conjunction with years of reporting documenting similar conditions for incarcerated individuals in disasters merits drastic change. Yet, a scoping review found that improved internal policies and emergency management initiatives alone is insufficient to deal with increasing environmental hazards and the particular vulnerability of the incarcerated population (Le Dé & Gaillard, 2017). Emergency management plans from states and counties, not only prisons and jails, must include carceral facilities and facilities themselves must also take measures to ensure safety (e.g., regulations creating maximum numbers for individuals in cells).

Lastly, this work further positions the legal system as one that is integral to our understanding of how and for whom risk is socially produced during climate disasters (Wisner, B et al., 2003). It also emphasizes that both physical and mental health is a critical component of this risk that cannot be left out. And, it adds to the recent and growing literature (Pellow, 2024) that disasters increase the risk of poor mental health outcomes in prisons and jails. This is because disasters increase stress, anxiety, and fear – likely due to further reduced agency, access to information, and the particularly abhorrent conditions that individuals experience while incarcerated during a disaster.

This work has limitations to note. First, individuals were not representative, as they were selected based on their accessing syringe services. However, this analysis was meant to serve as a descriptive first step in better understanding the experience of hurricanes for incarcerated individuals, unveiling how climate disasters disproportionately affect individuals both during and post-incarceration and the lack of preparedness of facilities during such events, which points to critical areas for future study.

In conclusion, climate disasters impose additional trauma on individuals already living in stressful, unpredictable environments. We add to literature at the intersection of the criminal legal system and disasters by highlighting critical health impacts and providing evidence to further conceptualize carceral spaces as ones particularly at risk of poor health outcomes during disasters. Work at the intersection of the criminal legal system and health must consider how the climate crisis affects this relationship and how the collateral consequences of incarceration for housing, employment, finances, and other basic needs further disadvantage this population during disasters.
